# Supplementary material for: Treatment with Beta-Blockers and ACE-Inhibitors in Breast Cancer Patients Receiving Adjuvant Trastuzumab-Based Therapy and Developing Mild Cardiac Toxicity: A Prospective Study
Source: Cancers (Basel). 2020 Jan 31;12(2):327. doi: 10.3390/cancers12020327 (PMC7072182; doi:10.3390/cancers12020327)
Supplement: Supplementary file 1 [file cancers-12-00327-s001.pdf]

cancers

Article

Treatment with Beta-Blockers and ACE-Inhibitors in Breast Cancer Patients Receiving Adjuvant Trastuzumab-Based Therapy and Developing Mild Cardiac Toxicity: A Prospective Study

Supplementary Material

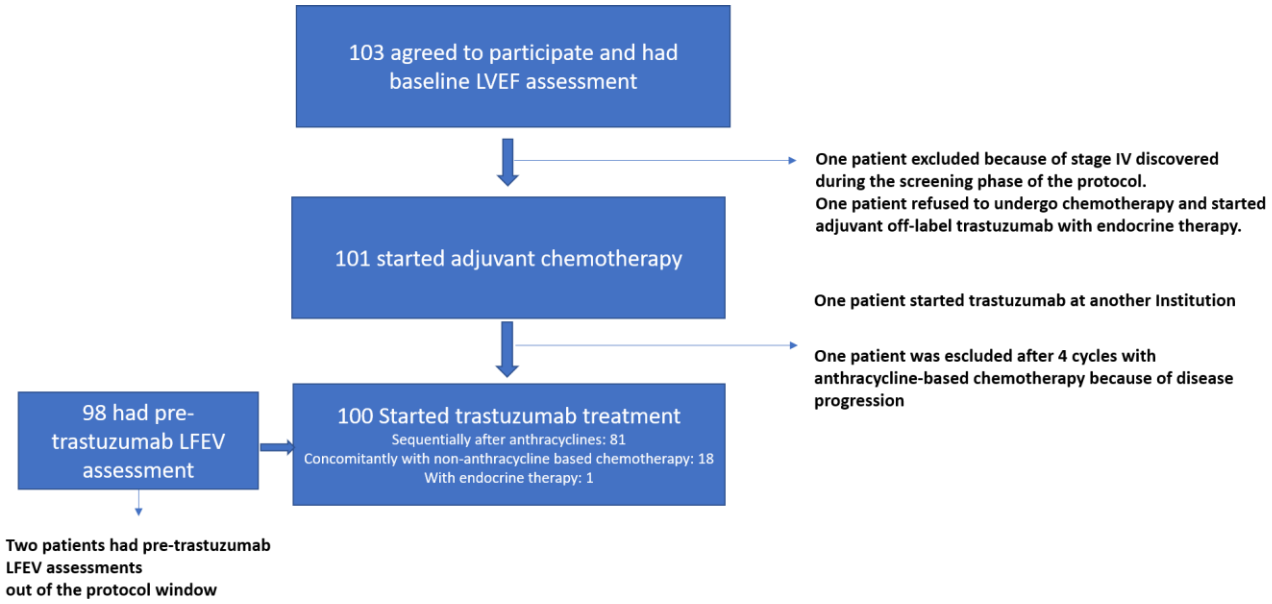

Figure S1. Diagram of Patient's Flow in the Trial.

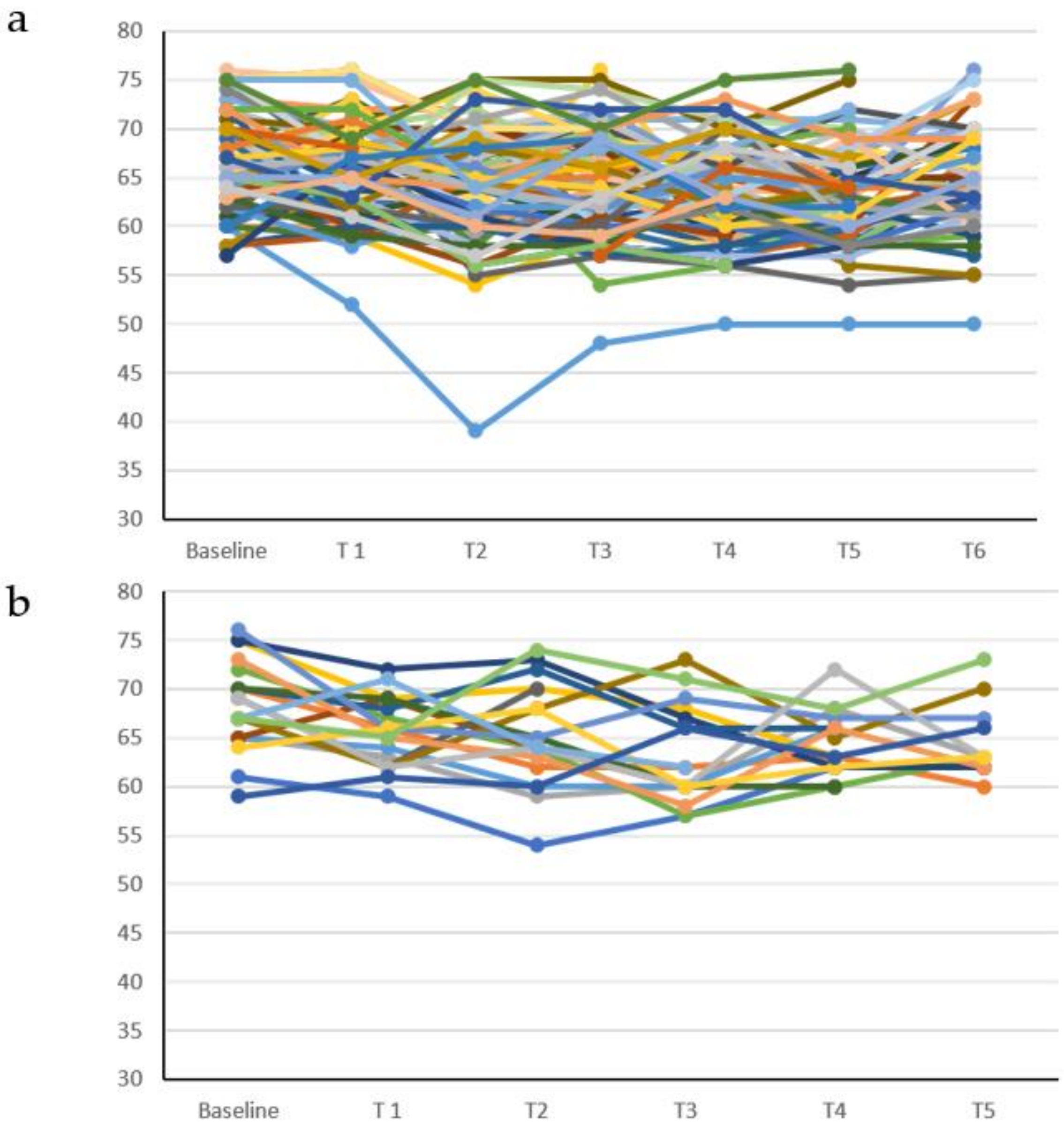

Figure S2. Individual LVEF determinations during the study according to receipt of anthracycline. Panel **a**: Individual LVEF values in patients receiving anthracycline. Panel **b**: Individual LVEF values in patients not receiving anthracycline.

a

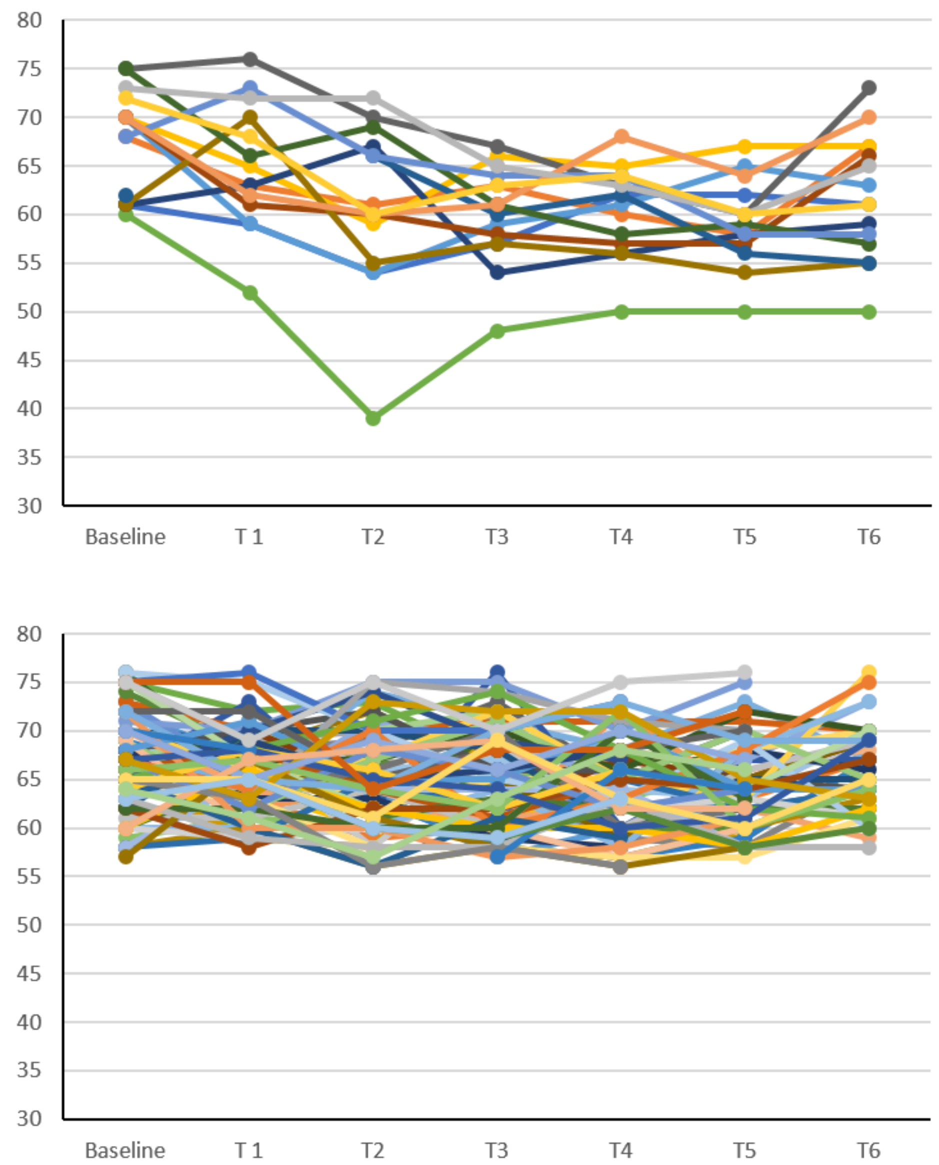

Figure S3. Individual LVEF determinations during the study in patients developing and not developing the cardiac event of interest. Panel **a**; Individual LVEF values in patients developing the cardiac event of interest. Panel **b**; Individual LVEF values in patients not developing the cardiac event of interest.

Table S1. Summary of LVEF findings.

Timepoint	N of patients	Median Value (%)	95% C.I.	Range
Baseline (overall)	103	67	66–68	57–76
Baseline in group 1*	81	67	66–68	57–76
Baseline in group 2*	19	69	66–71	59–76
Pre-trastuzumab (overall)	98	66	65–67	52–76
Pre-tratuzumab in group 1	79	66	65–67	52–76
Nadir LVEF (overall)	100	60	59–61	39–70
Nadir LFEV in group 1	81	60	59–61	39–70
Nadir LFEF in group 2	19	60	59–62	54–66
Post-treatment LVEF**	100	65	64–66	51–76
Post-treatment LVEF in group 1	81	65	64–67	51–76
Post-treatment LVEF in group 2	19	63	62–66	60–70

*Group 1; adjuvant anthracyclines followed by concomitant taxanes and trastuzumab; group 2; upfront adjuvant trastuzumab and chemotherapy or endocrine therapy (1 patient); **6 months to one year after trastuzumab completion.

Table S2. Summary of LVEF findings according to development cardiac events of interest.

Timepoint	Median Value (%)	95% C.I.	Range
Baseline, no events	67	66–68	57–76
Baseline, events	70	65–71	60–75
Pre-trastuzumab, no events	67	65–67	58–76
Pre-tratuzumab, events	66	62–69	52–76
Nadir LVEF, no events	60	60–62	56–70
Nadir LFEV, events	58	53–59	39–60
Post-trastuzumab* LVEF, no events	65	56–76	56–76
Post-trastuzumab* LVEF, events	64	61–67	51–73
Median LVEF recovery from Nadir; no events	4	4–5	0–16
Median LVEF recovery from Nadir: events	8	6–10	1–13

*6 months to one year after trastuzumab completion.
